# RNA-Guided Genome Editing for Target Gene Mutations in Wheat

**DOI:** 10.1534/g3.113.008847

**Published:** 2013-10-11

**Authors:** Santosh Kumar Upadhyay, Jitesh Kumar, Anshu Alok, Rakesh Tuli

**Affiliations:** National Agri-Food Biotechnology Institute, Department of Biotechnology, Government of India, Mohali, Punjab, India 160071

**Keywords:** Cas, cgRNA, CRISPR, genome editing, indel, wheat genome editing

## Abstract

The clustered, regularly interspaced, short palindromic repeats (CRISPR) and CRISPR-associated protein (Cas) system has been used as an efficient tool for genome editing. We report the application of CRISPR-Cas–mediated genome editing to wheat (*Triticum aestivum*), the most important food crop plant with a very large and complex genome. The mutations were targeted in the *inositol oxygenase* (*inox*) and *phytoene desaturase* (*pds*) genes using cell suspension culture of wheat and in the *pds* gene in leaves of *Nicotiana benthamiana*. The expression of chimeric guide RNAs (cgRNA) targeting single and multiple sites resulted in indel mutations in all the tested samples. The expression of Cas9 or sgRNA alone did not cause any mutation. The expression of duplex cgRNA with Cas9 targeting two sites in the same gene resulted in deletion of DNA fragment between the targeted sequences. Multiplexing the cgRNA could target two genes at one time. Target specificity analysis of cgRNA showed that mismatches at the 3′ end of the target site abolished the cleavage activity completely. The mismatches at the 5′ end reduced cleavage, suggesting that the off target effects can be abolished *in vivo* by selecting target sites with unique sequences at 3′ end. This approach provides a powerful method for genome engineering in plants.

Specific and effective genome editing through nontransgenic approaches is an area of high-priority research for the improvement of food crops. Several genome editing technologies like zinc finger nuclease (ZFN) and transcription activator–like effector nuclease (TALEN) have been deployed for targeted genome modifications (Chen and Gao 2013; Zhang *et al.* 2010), but these are rather complicated in design and need protein engineering for each target sequence. Recently, a new technology based on the type II prokaryotic clustered, regularly interspaced, short palindromic repeats (CRISPR) and CRISPR-associated protein (Cas) system has been developed as an effective tool for genome engineering (Cong *et al.* 2013; Mali *et al.* 2013). It is highly specific, inexpensive, and easy to engineer. CRISPR consists of an array of repeat sequences separated by “spacer” sequences that belong to the targeted gene/genome. A long primary transcript transcribes from CRISPR arrays and gets processed into short CRISPR RNAs (crRNAs). The crRNA consists of a conserved repeat sequence and a variable spacer sequence (guide) complementary to the target gene sequence (Brouns *et al.* 2008; Hale *et al.* 2009). Trans activating crisper RNA (tracer RNA) is another important molecule that plays a critical role in the processing of pre-crRNA (Chylinski *et al.* 2013). It is a short RNA sequence and is complementary to the CRISPR repeat. It activates the processing and maturation of pre-crRNA into the short crRNA by RNAseIII and Cas9. The ribonucleoprotein complex formed by short crRNA and Cas9 proteins binds to the target sequence by base pairing (Jore *et al.* 2011) and causes sequence-specific dsDNA cleavage. A chimeric crRNA and tracer RNA hybrid has also been designed and has been reported to be as effective as those used individually (Mali *et al.* 2013). The presence of a conserved sequence motif (NGG) known as protospacer adjacent motif (PAM) at 3′ downstream of target spacer sequence is also reported as essential for cleavage (Gasiunas *et al.* 2012). The CRISPR-Cas system has been demonstrated to work efficiently for genome editing in bacterial, yeast, and animal systems (Cong *et al.* 2013; DiCarlo *et al.* 2013; Gasiunas *et al.* 2012; Jinek *et al.* 2013; Mali *et al.* 2013) and has been applied to plants recently (Li *et al.* 2013; Nekrasov *et al.* 2013; Shan *et al.* 2013). Although, some nonspecific editing has been reported (Fu *et al.* 2013), the CRISPR-Cas system is very simple to design, highly effective (Mali *et al.* 2013; Cong *et al.* 2013), and can be improved in specificity.

We report the application of the CRISPR-Cas–mediated genome editing in wheat (*Triticum aestivum*), the most important food crop plant, and *Nicotiana benthamiana*, a model plant species. Mutations in the *inositol oxygenase* (*inox*) and *phytoene desaturase* (*pds*) genes in cell suspension culture of wheat and the *pds* gene in leaves of *N. benthamiana* were targeted. The efficacy of multiplexed CRISPR RNA, targeting two different positions in two separate genes, was also studied.

## Materials and Methods

### Synthesis of chimeric guide RNA encoding DNAs

We targeted the *inox* and *pds* genes of wheat and the *pds* gene of *N. benthamiana* to demonstrate RNA-guided genome editing in plants. Partial gene sequences were amplified from genomic DNA of the wild-type plants. Target sites (also known as protospacer) of 20 nucleotides were selected manually (Supporting Information, File S1) following the criteria described previously (Hwang *et al.* 2013; Mali *et al.* 2013). The presence of NGG trinucleotide protospacer adjacent motif (PAM) at the 3′ end of the target region was an essential criterion in target selection. The chimeric guide RNAs (cgRNAs) were designed to target one or two sites in the targeted genes (Hwang *et al.* 2013; Mali *et al.* 2013) (File S2). Overlapping primers were used for the synthesis of cgRNAs transcribing DNA. To develop duplex cgRNA for targeting two regions at one time, two cgRNAs were fused by using the *Spe*I restriction site. Assembled DNA was amplified by end primers, cloned, and sequenced.

### Construction of expression vectors

**Figure 1:**
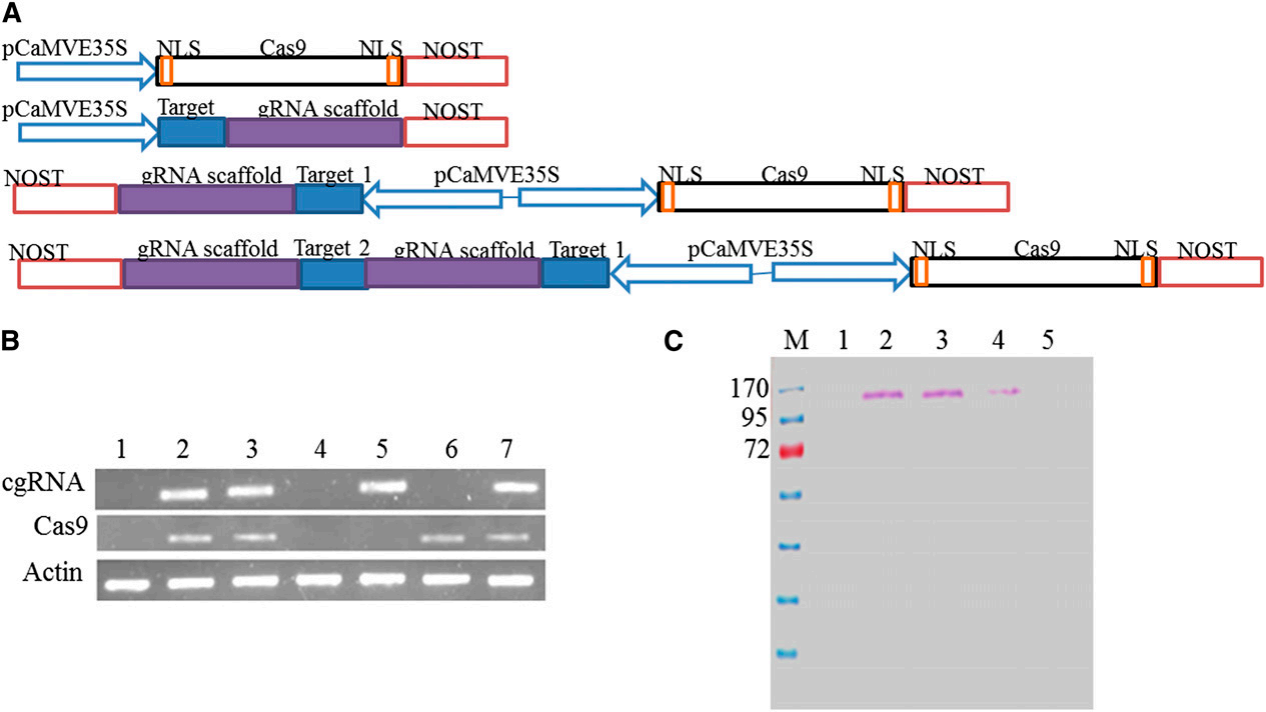
Basic architecture of constructs used for CRISPR-Cas–mediated genome editing and expression analysis of cgRNA and Cas9. (A) Architecture of constructs used for expression. The four types of constructs developed for the expression of cgRNA and Cas9 are shown. (B) RT-PCR analysis of expression of cgRNA and Cas9 in wheat: untransformed (lane 1); pCinox1-transformed (lane 2) suspension cells; and pCwpds1-transformed (lane 3) suspension cells RT-PCR analysis of expression of cgRNA and Cas9 in. *N. benthamiana*: untransformed (lane 4); pTpds1 (lane 5); pCas9-transformed (lane 6) leaf; and pCtpds1-transformed (lane 7) leaf. (C) Western blot analysis of Cas9 using anti-Flag antibody (FLAG tag attached at N-terminus of Cas9). Lane M, molecular weight marker; lane 1, untransformed; lane 2, pCinox1-transformed suspension cells of wheat; lane 3, pCwpds1-transformed suspension cells of wheat; lane 4, pCtpds1 agro-infiltrated; lane 5, untransformed *N. benthamiana* leaves.

Four types of expression vectors were prepared by cloning *cgRNA* and *Cas9* in different combinations (Figure 1A). Constitutive CaMVE35S promoter was used to drive the expression. We cloned *cgRNA* and *Cas9* alone, as well as together, in plant expression vector pBI121. The *Cas9* gene (4272 bp) including the FLAG tag and nuclear localization signal for eukaryotic expression was obtained from Addgene USA (Addgene plasmid 42229). The plant expression constructs and their target genes are provided in Table S1.

### Plant transformation

*Agrobacterium tumefaciens* strain GV3101 was used for genetic transformation of *N. benthamiana* and wheat cells. Transient expression of cgRNA and Cas9 in different combinations in 3-wk-old to 4-wk-old *N. benthamiana* plant leaves was performed by agro-infiltration, as described by Bos *et al.* (2006). The infiltrated area was marked and used for DNA and RNA isolation 4 d after infiltration.

To analyze the mutations in wheat, cell suspension culture developed from immature embryos was used for transformation following the protocol of Wu *et al.* (1998). Transformed cells were enriched by antibiotic selection and used for the isolation of DNA, RNA, and protein.

### RT-PCR for expression analysis of cgRNA and Cas9

Total RNA was isolated from the transformed wheat suspension cells and agro-infiltrated area of *N. benthamiana* leaves, and was used to make cDNA. PCR amplification was performed with the primers listed in File S1 for the analysis of cgRNA expression. Cas9 expression was analyzed by using primers Cas9 forward 5′ tctgtgggctgggccgtgatc 3′ and Cas9 reverse 5′ cagatccggttcttccgtctgg 3′. Amplification of *actin* was used as control.

### Western blotting

Total soluble protein was extracted from transformed wheat suspension cells and *N. benthamiana* leaves using the extraction buffer (20 mM Tris pH 8, 150 mM NaCl, 0.05% TritonX-100, and 100 µM PMSF), separated on 10% SDS-PAGE, and transferred onto PVDF membrane. Cas9 protein was detected by using anti-FLAG antibody (FLAG tag was attached at N-terminus of Cas9).

### Mutation analysis

Mutations in targeted genes in plants were analyzed by PCR amplification and Sanger sequencing. Genomic DNA was isolated from transformed tissue and used as template in amplification. Primers from the vicinity of the targeted regions (highlighted in green in File S1) were used for amplification of control and transformed samples. Ten cycles of amplification were performed. The amplified DNA was cloned and used for sequencing. Sequences were aligned against wild-type, and average mutation percentage was calculated from three independent replicates. A minimum of 75 clones were sequenced for each transforming event and used for the analysis.

### Specificity analysis of cgRNA

To analyze the specificity of cgRNA in binding to the target region, several sets of mutations were made in protospacer 1 binding site of *inox* cgRNA (File S1 and Table S2). Mutant cgRNA constructs were transformed with Cas9. Their editing potential was analyzed by amplification with the aforementioned primers and BsgI digestion (BsgI restriction site is present at target region in *inox* protospacer 1) (File S1). Mutated DNA could not be digested with BsgI. Average mutation percentage was estimated by densitometry.

## Results

### Target selection, construction of expression cassettes, and expression of Cas9 and cgRNAs

Despite using the expression of complete crRNA and tracer RNA, we designed *cgRNA* for gene targeting as described previously (Hwang *et al.* 2013; Mali *et al.* 2013). This reduces the complexity of the system. The cgRNA consisted of mature crRNA fused with partial tracer RNA, which mimics the natural architecture of crRNA and tracer RNA duplex. We used an 80-nucleotides-long guide RNA scaffold sequences fused with 20-nucleotide target (spacer) sequences at 5′ end (File S2).

We targeted two regions in each gene, separately and together. Therefore, several combinations of expression constructs were developed (Table S1); a representative architecture is shown in Figure 1A. Separate expression cassettes were developed for Cas9 (pCas9) and cgRNA (pTpds1 and pTpds2) to target the *pds* gene in *N. benthamiana* and transformed into leaves by agro-infiltration in different combinations (pCas9 or pTpds alone or together in 1:1 ratio). No mutation was detected when Cas9 and cgRNA were taken, one at a time, as expected. Combined expression of pCas9 and pTpds in 1:1 ratio provided mutations that increased significantly when Cas9 and cgRNA were coexpressed using the construct pCtpds1 (Table 1).

**Table 1:** Average mutation rate at protospacer 1 of *phytoene desaturase* gene of *Nicotiana benthamiana* after agro-infiltration with different constructs

Construct Used	Average Mutation (%) at Target Sites
pCas9	0
pTpds1	0
pCas9 and pTpds1 (1:1 ratio)	1.8 (2/108)
pCtpds1	12.7 (12/94)

RT-PCR of total RNA from transformed cells showed the expression of cgRNA and Cas9 in cells of both plants (Figure 1B). Cas9 protein was also detected by Western blotting using anti-FLAG antibody against the FLAG tag attached at N-terminus (Figure 1C).

### cgRNA-Cas9 guided genome editing in *N. benthamiana*

**Figure 2:**
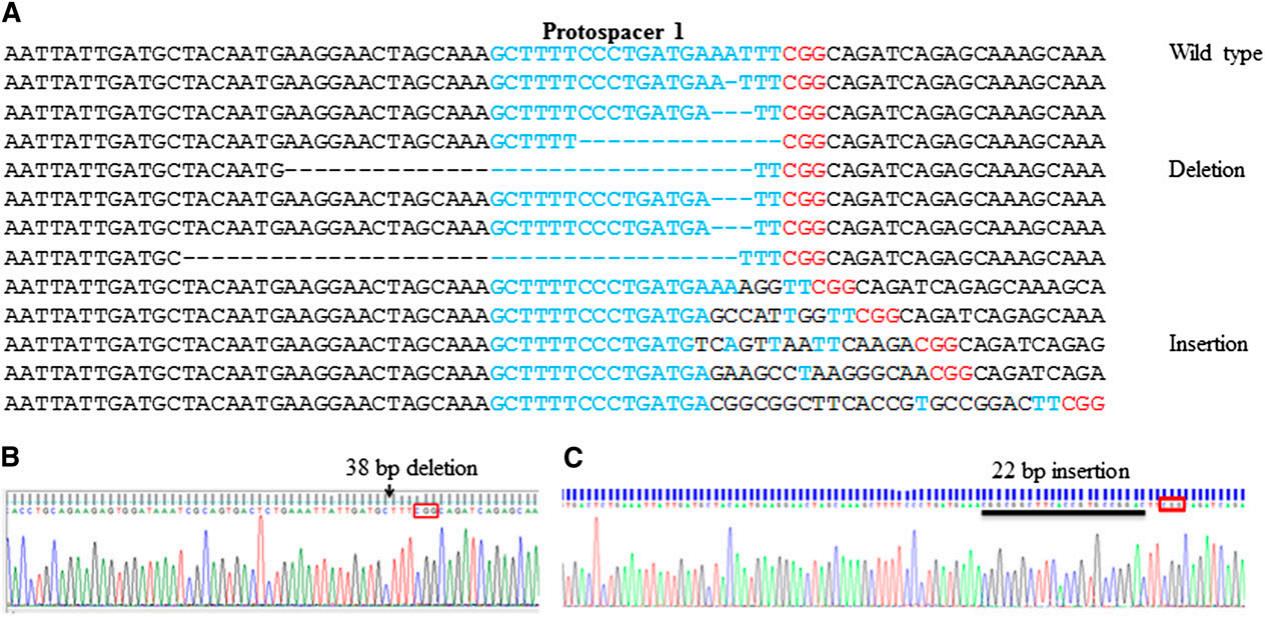
Editing at protospacer 1 of *phytoene desaturase* (*pds*) gene of *N. benthamiana* by the CRISPR-Cas system. (A) Alignment of wild-type and sequences with indel at protospacer 1 of the *pds* gene. (B) and (C) Sanger sequencing of selected deletion and insertion mutants, respectively.

As described, we targeted two sites in the *pds* gene of *N. benthamiana* leaves by agro-infiltration (File S1). We detected mutations (deletions and insertions) in *N. benthamiana* in 1.8% of the clones obtained from leaves infiltrated with the two *Agrobacterium* strains (containing pCas9 or pTpds1) mixed in 1:1 ratio. However, 12.7% indels were detected in *N. benthamiana* when Cas9 and cgRNA were present within a single plasmid construct pCtpds1 (Table 1) and, hence, codelivered. In protospacer 2 region, 2.4% and 13.8% indels were detected with pCas9:pTpds2 (1:1) and pCtpds2 treatments, respectively. The results established that the co-delivery of cgRNA and Cas9 improved efficacy of the system. Therefore, in case of wheat only, co-delivery was used to examine the application of the CRISPR-Cas system. A maximum of 38 bp deletion and of 22 bp addition were detected (Figure 2). The indel pattern was similar to that reported in other studies (Hwang *et al.* 2013; Li *et al.* 2013; Mali *et al.* 2013; Nekrasov *et al.* 2013; Shan *et al.* 2013). Maximum indels were noticed approximately 2–3 bp away from the 5′ end of PAM.

### cgRNA-Cas9 guided genome editing in *T. aestivum*

**Figure 3:**
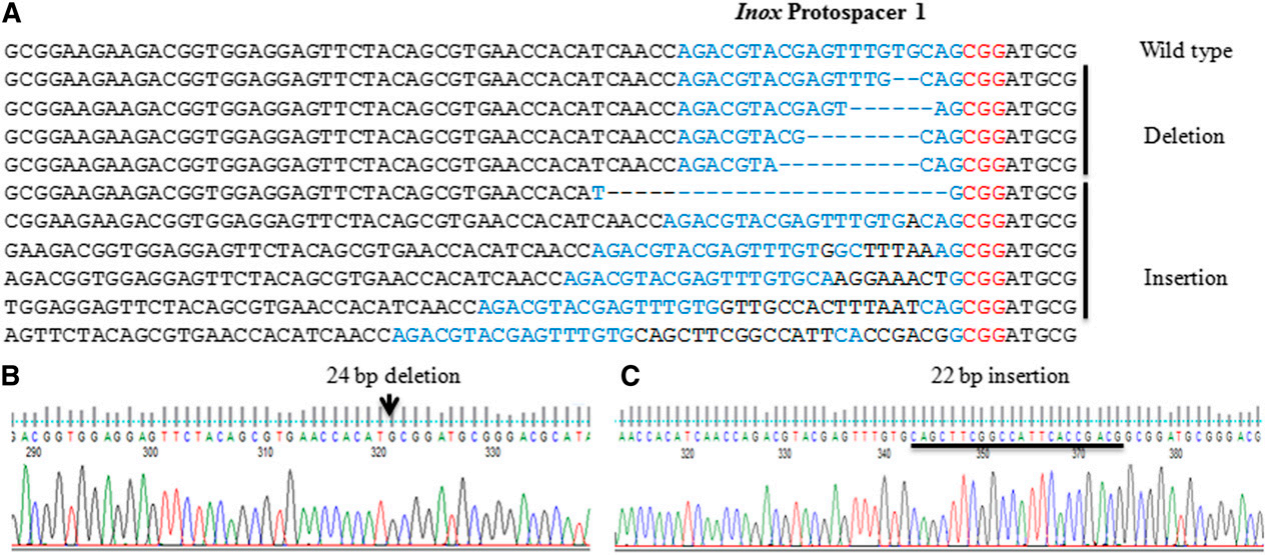
CRISPR-cas–guided editing at protospacer 1 of *inositol oxygenase* (*inox*) gene of *T. aestivum*. (A) Alignment of wild-type and sequences with indel at protospacer 1 of *inox* gene. (B) and (C) Sanger sequencing of selected deletion and insertion mutants, respectively.

Four regions of the two genes (*inox* and *pds*) were targeted for editing in suspension cells of wheat (File S1). The rate of mutations observed in wheat was higher as compared to that in *N. benthamiana*. We observed 18–22% mutations in different protospacers (Table 2) in wheat. Both deletion and insertion mutations were detected. A maximum of 24 bp deletion and of 22 bp insertion were detected in *inox* protospacer 1 (Figure 3). Indels were located at similar positions (2–3 bp away from the 5′ end of PAM) in wheat, as observed in *N. benthamiana* and as reported in other studies (Hwang *et al.* 2013; Li *et al.* 2013; Mali *et al.* 2013; Nekrasov *et al.* 2013; Shan *et al.* 2013).

**Table 2:** Average indel percentage in *T. aestivum* at different protospacers with related expression cassettes

Expression Cassette	Indel (%)	
	Protospacer 1	Protospacer 2
pCinox1	17.9 (14/78)	NA
pCinox2	NA	20.7 (17/82)
pCwpds1	22.3 (19/85)	NA
pCwpds2	NA	18.4 (14/76)
pCinox12	12.7 (12/94)	11.2 (9/80)
pCpin1	10.2 (8/78)	8.6 (7/81)

NA, not applicable.

### Duplex cgRNA-Cas9 induced deletion in *inox* gene

**Figure 4:**
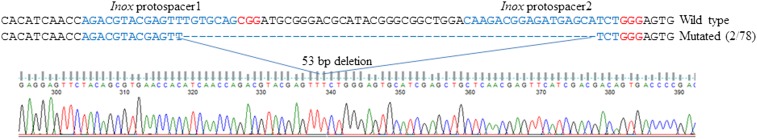
Duplex cgRNA-Cas9–induced deletion in *inox* gene. The figure shows that the targeting of two protospacers in one gene resulted into the deletion of the complete fragment between targeted regions.

To analyze the efficacy of duplex cgRNA targeting two sites simultaneously in one gene, we fused the two cgRNA (pCinox12) targeting the *inox* gene in wheat. The expression of duplex cgRNA caused indels at both the sites. However, the efficiency was lower as compared to that of the single cgRNA (Table 2). Approximately 11–12% of mutations were detected at each site. Complete deletion of the region between two targeted sites was observed in 2.8% cases (Figure 4).

### Multiple gene targeting with cgRNA-Cas9

To detect the editing potential of cgRNA-Cas9 for more than one gene at a time, we fused cgRNA targeting the protospacer 1 of *pds* and *inox* genes of wheat (pCpin1). Expression of this cassette in suspension cells caused indels at both the target sites, although with different efficiency (Table 2). Approximately 10% of indels were detected in *pds* and 8% were detected in *inox* gene.

### Target specificity of cgRNA

To evaluate the off-target effects of CRISPR, several sets of random mutations in protospacer 1 binding site of *inox* cgRNA were developed (Table S2) and transformed in suspension cells of wheat for coexpression with Cas9. The mutations at the 3′ end of the target binding region abolished the genome cleavage activity completely in the presence of a single base mismatch up to base 12. Multiple mutations up to the base 16 position also prevented cleavage (Figure S1), whereas single base mismatches at the 5′ end were tolerated to some extent and provided 1–3% mutation.

## Discussion

The study demonstrates that the CRISPR-Cas system can be applied successfully to genome editing in very large genomes such as the 17 Gb hexaploid genome of wheat. The application of the CRISPR-Cas system has been demonstrated in bacteria, yeast, and animal cells with a high percentage of indels (Cong *et al.* 2013; Mali *et al.* 2013; DiCarlo *et al.* 2013; Jinek *et al.* 2013). Application of this system in plants has also been reported using transient expression by protoplast transformation and agro-infiltration (Li *et al.* 2013; Nekrasov *et al.* 2013; Shan *et al.* 2013).

We have demonstrated that CRISPR-Cas–guided genome editing can be achieved by using wheat suspension cells. This makes the application of this technique simpler to crop plants that can be regenerated from cell cultures. We also showed that the deletion of a gene fragment can be achieved in wheat by simultaneous cleavage at two targeted sites within one gene. Similar deletion has been reported in the case of *Arabidopsis* (Li *et al.* 2013; Mao *et al.* 2013), although its genome size is much smaller than the wheat genome. This result suggests that the method can be useful in deletion of target regions from the genome and the development of knockout mutants.

We have also analyzed the efficacy of multiplex cgRNA targeting two genes at one time. Significant indels were detected in both the target genes in wheat. Because the experiment was performed with suspension cells, we cannot comment on whether both the target sites were mutated in the same cell or in different cells. However, the results establish that the method introduces mutations in multiple genes using the same expression cassette. Such results have been reported in the case of animal systems (Cong *et al.* 2013) but have not been reported in plants.

The CRISPR-Cas system is much simpler than the other reported systems such as ZFN and TALEN (Chen and Gao 2013; Zhang *et al.* 2010). High-efficiency editing can be achieved even in large and complex plant genomes at each of the multiple targeted locations using the CRISPR-Cas system. Unlike ZFNs, it is not necessary to screen several cgRNAs to target the sites. Certain off-target mutations have also been reported (Fu *et al.* 2013) in the case of mismatch at the 5′ end of the target binding region. We observed that even single mismatch toward the 3′ end until base 12 abolished the binding of CRISPR completely. Similar results have been reported in the case of animal and bacterial systems (Cong *et al.* 2013). Although our experiments for the detection of off-target binding do not include extensive analysis, the results suggested that off-target mutations can be avoided by careful selection of the target sequences. Instead of targeting the conserved and semiconserved regions, unique sequences should be chosen during CRISPR design, especially toward the 3′ side.

Because of the simplicity in designing cgRNA (Cong *et al.* 2013) and the applicability to a wide variety of plants and animals, the CRISPR-Cas system will be a very useful tool for reverse genetics as well as functional genomics. The necessity of NGG (PAM) at the 3′ of the target region is the only limitation for the target site.

## Supplementary Material

Supporting Information

## References

[bib1] Brouns S. J. Jore M. M. Lundgren M. Westra E. R. Slijkhuis R. J., 2008 Small CRISPR RNAs guide antiviral defense in prokaryotes. Science 321: 960–964. 18703739 10.1126/science.1159689 PMC5898235

[bib2] Bos J. I. B. Kanneganti T. D. Young C. Cakir C. Huitema E., 2006 The C-terminal half of *Phytophthora infestans* RXLR effector AVR3a is sufficient to trigger R3a-mediated hypersensitivity and suppress INF1-induced cell death in *Nicotiana benthamiana*. Plant J. 48: 165–176. 16965554 10.1111/j.1365-313X.2006.02866.x

[bib3] Chen K. Gao C., 2013 TALENs: Customizable molecular DNA scissors for genome engineering of plants. J. Genet. Genomics 40: 271–279. 23790626 10.1016/j.jgg.2013.03.009

[bib4] Chylinski K. Le R. A. Charpentier E., 2013 The tracrRNA and Cas9 families of type II CRISPR-Cas immunity systems. RNA Biol. 10: 1–12. 23563642 10.4161/rna.24321 PMC3737331

[bib5] Cong L. Ran F. A. Cox D. Lin S. Barretto R., 2013 Multiplex genome engineering using CRISPR/Cas systems. Science 339: 819–823. 23287718 10.1126/science.1231143 PMC3795411

[bib6] DiCarlo J. E. Norville J. E. Mali P. Rios X. Aach J., 2013 Genome engineering in *Saccharomyces cerevisiae* using CRISPR-Cas systems. Nucleic Acids Res. 41: 4336–4343. 23460208 10.1093/nar/gkt135 PMC3627607

[bib7] Fu Y. Foden J. A. Khayter C. Maeder M. L. Reyon D., 2013 High-frequency off-target mutagenesis induced by CRISPR-Cas nucleases in human cells. Nat. Biotechnol. 31: 822–826. DOI: 10.1038/nbt.2623. 10.1038/nbt.2623 PMC3773023 23792628

[bib8] Gasiunas G. Barrangou R. Horvath P. Siksnys V., 2012 Cas9-crRNA ribonucleoprotein complex mediates specific DNA cleavage for adaptive immunity in bacteria. Proc. Natl. Acad. Sci. U.S.A. 109. 109: E2579–E2586. 10.1073/pnas.1208507109 PMC3465414 22949671

[bib9] Hale C. R. Zhao P. Olson S. Duff M. O. Graveley B. R., 2009 RNA-guided RNA cleavage by a CRISPR RNA-Cas protein complex. Cell 139: 945–956. 19945378 10.1016/j.cell.2009.07.040 PMC2951265

[bib10] Hwang W. Y. Fu Y. Reyon D. Maeder M. L. Tsai S. Q., 2013 Efficient genome editing in zebrafish using a CRISPR-Cas system. Nat. Biotechnol. 31: 227–229. 23360964 10.1038/nbt.2501 PMC3686313

[bib11] Jinek M. East A. Cheng A. Lin S. Ma E., 2013 RNA-programmed genome editing in human cells. Elife 2: e00471. 23386978 10.7554/eLife.00471 PMC3557905

[bib12] Jore M. M. Lundgren M. Duijn E. V. Bultema J. B. Westra E. R., 2011 Structural basis for CRISPR RNA-guided DNA recognition by Cascade. Nat. Struct. Mol. Biol. 18: 529–536. 21460843 10.1038/nsmb.2019

[bib13] Li J. F. Norville J. E. Aach J. McCormack M. Zhang D., 2013 Multiplex and homologous recombination–mediated genome editing *in Arabidopsis* and *Nicotiana benthamiana* using guide RNA and Cas9. Nat. Biotechnol. 31: 688–691. DOI: 10.1038/nbt.2654. 10.1038/nbt.2654 PMC4078740 23929339

[bib14] Mali P. Yang L. Esvelt K. M. Aach J. Guell M., 2013 RNA-guided human genome engineering via Cas9. Science 339: 823–826. 23287722 10.1126/science.1232033 PMC3712628

[bib15] Nekrasov V. Staskawicz B. Weigel D. Jones J. D. J. Kamoun S., 2013 Targeted mutagenesis in the model plant *Nicotiana benthamiana* using Cas9 RNA-guided endonuclease. Nat. Biotechnol. 31: 691–693. DOI: 10.1038/nbt.2655. 10.1038/nbt.2655 23929340

[bib16] Shan Q. Wang Y. Li J. Zhang Y. Chen K., 2013 Targeted genome modification of crop plants using a CRISPR-Cas system. Nat. Biotechnol. 31: 686–688. DOI: 10.1038/nbt.2650. 10.1038/nbt.2650 23929338

[bib17] Wu H. McCormac A. C. Elliott M. C. Chen D. F., 1998 *Agrobacterium*-mediated stable transformation of cell suspension cultures of barley (*Hordeum vulgare*). Plant Cell Tissue Organ Cult. 54: 161–171.

[bib18] Zhang F. Maeder M. L. Unger-Wallace E. Hoshaw J. P. Reyon D., 2010 High frequency targeted mutagenesis in *Arabidopsis thaliana* using zinc finger nucleases. Proc. Natl. Acad. Sci. USA 107: 12028–12033. 20508152 10.1073/pnas.0914991107 PMC2900673

