# Supplementary material for: RNA-Guided Genome Editing for Target Gene Mutations in Wheat
Source: G3 (Bethesda). 2013 Oct 11;3(12):2233–8. doi: 10.1534/g3.113.008847 (PMC3852385; doi:10.1534/g3.113.008847)
Supplement: Supporting Information [file supp_3_12_2233__index.html]

RNA-Guided Genome Editing for Target Gene Mutations in Wheat — Supporting Information 

# RNA-Guided Genome Editing for Target Gene Mutations in Wheat

## Supporting Information for Upadhyay *et al.*, 2013

**Files in this Data Supplement:**

- Supporting Information - Figure S1, Files S1-S2, and Tables S1-S2 (PDF, 420 KB)
- Figure S1 - Sequence specificity analysis of CRISPR-Cas system by using different mutated cgRNAs. (PDF, 325 KB)
- File S1 - Partial sequence of (A) *inositol oxygenase* (*inox*) and (B) *phytoene desaturase* (*pds*) genes of wheat, and (C) *pds* gene of *Nicotiana benthamiana* used to decide the target for modification by CRISPR-Cas System. (PDF, 342 KB)
- File S2 - Chimeric guide RNA (cgRNA) for targeting (A) *inositol oxygenase* (*inox*) and (B) *phytoene desaturase* (*pds*) genes of wheat, and (C) *pds* gene of *Nicotiana benthamiana*. (PDF, 336 KB)
- Table S1 - Plant expression vectors used in the study. (PDF, 336 KB)
- Table S2 - Specificity analysis of cgRNA. (PDF, 304 KB)
