# Supplementary material for: RNA-Guided Genome Editing for Target Gene Mutations in Wheat
Source: G3 (Bethesda). 2013 Oct 11;3(12):2233–8. doi: 10.1534/g3.113.008847 (PMC3852385; doi:10.1534/g3.113.008847)
Supplement: Supporting Information [file supp_g3.113.008847_008847SI.pdf]

G3: Genes | Genomes | Genetics

RNA guided genome editing for target gene mutations in wheat

Santosh Kumar Upadhyay, Jitesh Kumar, Anshu Alok, RakeshTuli

National Agri-Food Biotechnology Institute
(Department of Biotechnology, Government of India)
C-127, Industrial Area, S.A.S. Nagar, Phase 8,
Mohali, Punjab, India-160071.

Corresponding author:
RakeshTuli
Executive Director
National Agri-Food Biotechnology Institute
(Department of Biotechnology, Government of India)
C-127, Industrial Area, S.A.S. Nagar, Phase 8,
Mohali, Punjab, India-160071.
Email: rakeshtuli@hotmail.com
Tel: +91 172 2290101
Fax: +91 172 4604888

DOI: 10.1534/g3.113.008847

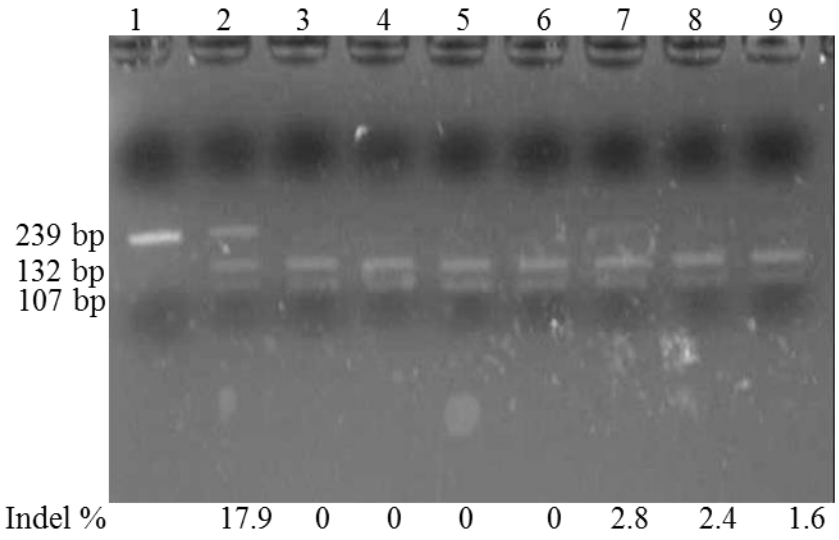

Figure S1 Sequence specificity analysis of CRISPR-Cas system by using different mutated cgRNAs. List of mutant cgRNA is given in Supplementary table 2. Normal and mutant cgRNAs (targeting protospacer 1 of *inox* gene) were co-expressed with Cas9 in suspension cells of *T. aestivum*. Targeted region was amplified, and digested with BsgI restriction enzyme (BsgI restriction site is present at cleavage position in *inox* protospacer1). BsgI could not digest the mutated DNA amplicon. Lane 1 undigested amplicon and lane 2-9 digested with BsgI. Lane 2, mutated with normal cgRNA; 3, wild type amplicon; 4-9, mutated with mutant cgRNA 1, 6, 9, 11, 12 and 13, respectively. Figure shows that mutations at 3' end of target binding region of cgRNA upto 12th bases completely abolished the cleavage activity. However mutations were detected in case of 5' end mutant cgRNA.

File S1

Partial sequence of (A) *inositol oxygenase* (*inox*) and (B) *phytoene desaturase (pds)* genes of wheat, and (C) *pds* gene of *Nicotiana benthamiana* used to decide the target for modification by CRISPR-Cas System. Targeted regions are shown in blue font and PAM in red. Primer used for amplification of gene after editing is shown in green.

(A)

```
                                                      Forward primer
GCTCGACGGCGGCTTCACCGTGCCGGACTCCAACGCCTTCGGCCACACCTTCAGGGACTACGACGCGGAGTCGGA BASE PAIRS
CGAGCTGCCGCCGAAGTGGCACGGCCTGAGGTTGCGGAAGCCGGTGTGGAAGTCCCTGATGCTGCGCCTCAGCCT 1 TO 75
                                                    Protospacer 1
GCGGAAGAAGACGGTGGAGGAGTTCTACAGCGTGAACCACATCAACCAGACGTACGAGTTTGTGCAGCGGATGCG BASE PAIRS
CGCCTTCTTCTGCCACCTCCTCAAGATGTCGCACTTGGTGTAGTTGGTCTGCATGCTCAAACACGTCGCCTACGC 76 TO 150
                      Protospacer 2
GGACGCATACGGGCGGCTGGACAAGACGGAGATGAGCATCTGGGAGTGCATCGAGCTGCTCAACGAGTTCATCGA BASE PAIRS
CCTGCGTATGCCCGCCGACCTGTTCTGCCTCTACTCGTAGACCCTCACGTAGCTCGACGAGTTGCTCAAGTAGCT 151 TO 225

CGACAGTGACCCCGACCTGGACATGCCGCAGATCGAGCACCTCCTCCAAACCGCCGAGGCCATCCGCAAGGACTA BASE PAIRS
GCTGTCACTGGGGCTGGACCTGTACGGCGTCTAGCTCGTGGAGGAGGTTTGGCGGCTCCGGTAGGCGTTCCTGAT 226 TO 300
                                                     Reverse primer
```

Yellow highlighted region is the site for BsgI used for digestion of amplified DNA.

(B)

```
                    Forward primer                                          Protospacer 1
GCTGAGCTTGGTATTAGTGATCGCTTGCAATGGAAGGAACACTCCATGATATTTGCCATGCCAAACAAACCAGGA BASE PAIRS
CGACTCGAACCATAATCACTAGCGAACGTTACCTTCCTTGTGAGGTACTATAAACGGTACGGTTTGTTTGGTCCT 1 TO 75
                                             Protospacer 2
GAATACAGCCGTTTTGATTTTCCAGAGACTTTGCCGGCGCCCTTAAATGGAGTGTGGGCCATACTGAAAAACAAT BASE PAIRS
CTTATGTCGGCAAAACTAAAAGGTCTCTGAAACGGCCGCGGGAATTTACCTCACACCCGGTATGACTTTTTGTTA 76 TO 150

GAAATGCTTACTTGGCCGGAGAAGGTGAAG  BASE PAIRS
CTTTACGAATGAACCGGCCTCTTCCACTTC  151 TO 180

                    Reverse primer
```

(C)

```
           Forward primer
     ------------------------>
GAATTGGTTTTTGCACCTGCAGAAGAGTGGATAAATCGCAGTGACTCTGAAATTATTGATGCTACAATGAAGGAA BASE PAIRS
CTTAACCAAAAACGTGGACGTCTTCTCACCTATTTAGCGTCACTGAGACTTTAATAACTACGATGTTACTTCCTT 1 TO 75
             Protospacer 1                                       Protospacer 2
CTAGCAAAGCTTTTCCCTGATGAAATTTCGGCAGATCAGAGCAAAGCAAAAATATTGAAGTATCATGTTGTCAAA BASE PAIRS
GATCGTTTCGAAAAGGGACTACTTTAAAGCCGTCTAGTCTCGTTTCGTTTTTATAACTTCATAGTACAACAGTTT 76 TO 150

ACTCCAAGGTCTGTTTATAAAACTGTGCCAGGTTGTGAACCCTGCCGGCCCTTGCAAAGATCCCCTATAGAGGGG BASE PAIRS
TGAGGTTCCAGACAAATATTTTGACACGGTCCAACACTTGGGACGGCCGGGAACGTTTCTAGGGGATATCTCCCC 151 TO 225
                                    <-------------------
                                       Reverse primer
```

File S2

Chimeric guide RNA (cgRNA) for targeting (A) *inositol oxygenase* (*inox*) and (B) *phytoene desaturase (pds)* genes of wheat, and (C) *pds* gene of *Nicotiana benthamiana.* (i) cgRNA for protospacer 1, (ii) cgRNA for protospacer 2 and (iii) cgRNA for targeting both protospacer 1 and 2. Target region is given in blue font and guide RNA scaffold in red font. Restriction site SpeI (highlighted in yellow) was used for ligation of the two cgRNAs.

(A)

(i)

NNNNNAGACGTACGAGTTTGTGCAGGTTTTAGAGCTAGAAATAGCAAGTTAAAATAAGGCTA
GTCCGTTATCAACTTGAAAAAGTGGCACCGAGTCGGTGCTTTTNNNNNNNN

(ii)

NNNNNCAAGACGGAGATGAGCATCTGTTTTAGAGCTAGAAATAGCAAGTTAAAATAAGGCTA
GTCCGTTATCAACTTGAAAAAGTGGCACCGAGTCGGTGCTTTTNNNNNN

(iii)

NNNNNAGACGTACGAGTTTGTGCAGGTTTTAGAGCTAGAAATAGCAAGTTAAAATAAGGCTA
GTCCGTTATCAACTTGAAAAAGTGGCACCGAGTCGGTGCTTTACTAGTNNNNNCAAGACGGAG
ATGAGCATCTGTTTTAGAGCTAGAAATAGCAAGTTAAAATAAGGCTAGTCCGTTATCAACTT
GAAAAAGTGGCACCGAGTCGGTGCTTTTNNNNNN

(B)

(i)

NNNNNTTTGCCATGCCAAACAAACCGTTTTAGAGCTAGAAATAGCAAGTTAAAATAAGGCTA
GTCCGTTATCAACTTGAAAAAGTGGCACCGAGTCGGTGCTTTTNNNNNNNN

(ii)

NNNNNGGCGCCCTTAAATGGAGTGTGTTTTAGAGCTAGAAATAGCAAGTTAAAATAAGGCTA
GTCCGTTATCAACTTGAAAAAGTGGCACCGAGTCGGTGCTTTTNNNNNN

(C)

(i)

NNNNNGCTTTTCCCTGATGAAATTTGTTTTAGAGCTAGAAATAGCAAGTTAAAATAAGGCTA
GTCCGTTATCAACTTGAAAAAGTGGCACCGAGTCGGTGCTTTTNNNNNNNN

(ii)

NNNNNATCATGTTGTCAAAACTCCAGTTTTAGAGCTAGAAATAGCAAGTTAAAATAAGGCTA
GTCCGTTATCAACTTGAAAAAGTGGCACCGAGTCGGTGCTTTTNNNNNN

Table S1 Plant expression vectors used in the study.

Vector	Detail	Target gene
pCas9	Expression of FLAG tag and NLS containing Cas9 protein.	NA
pTpds1	Expression of cgRNA targeting protospacer 1 of *Nicotiana benthamiana phytoene desaturase (pds)* gene	*N. benthamiana pds* gene
pTpds2	Expression of cgRNA targeting protospacer 2 of *N. benthamiana pds* gene	*N. benthamiana pds* gene
pCtpds1	Co-expression of Cas9 and cgRNA targeting protospacer 1 of *N. benthamiana pds* gene	*N. benthamiana pds* gene
pCtpds2	Co-expression of Cas9 and cgRNA targeting protospacer 2 of *N. benthamiana pds* gene	*N. benthamiana pds* gene
pCinox1	Co-expression of Cas9 and cgRNA targeting protospacer 1 of *T. aestivum inositol oxygenase* (*inox*) gene	*T. aestivum inox* gene
pCinox2	Co-expression of Cas9 and cgRNA targeting protospacer 2 of *T. aestivum inox* gene	*T. aestivum inox* gene
pCwpds1	Co-expression of Cas9 and cgRNA targeting protospacer 1 of *T. aestivum pds* gene	*T. aestivum pds* gene
pCwpds2	Co-expression of Cas9 and cgRNA targeting protospacer 2 of *T. aestivum pds* gene	*T. aestivum pds* gene
pCinox12	Co-expression of Cas9 and duplex cgRNAs targeting protospacer 1 and 2 of *T. aestivum pds* gene	*T. aestivum pds* gene
pCpin1	Co-expression of Cas9 and duplex cgRNAs targeting protospacer 1 of both *inox* and *pds* gene of *T. aestivum.*	*T. aestivum inox* and *pds* gene

Table S2 Specificity analysis of cgRNA. Table shows average mutation percentage at targeted locus with different mutant cgRNA.

		Sequence	Average mutation (%)
Normal cgRNA		AGACGTACGAGTTTGTGCAG	17.9
Mutant cgRNA	1	AGACGTACGAGTTTGTGCAA	0
	2	AGACGTACGAGTTTGTGCGA	0
	3	AGACGTACGAGTTTGTGGCA	0
	4	AGACGTACGAGTTTGGTGCA	0
	5	AGACGTACGGAGTGCAGTTT	0
	6	AGACGTACTAGTTTGTGCAG	0
	7	AGACGTACGAGTTTGTGAAG	0
	8	AGACGTACGAGTTTGTCTAG	0
	9	AGACGTACAAGTTTATGCAG	0
	10	AGACGTACGAGTGGGTGCAG	0
	11	TGACGTACGAGTTTGTGCAG	2.8
	12	CAACGTACGAGTTTGTGCAG	2.4
	13	CAGCGTACGAGTTTGTGCAG	1.6
	14	AGACAGTCGAGTTTGTGCAG	0
	15	AGACTGCAGAGTTTGTGCAG	0
