# Supplementary material for: RNA-Guided Genome Editing for Target Gene Mutations in Wheat
Source: G3 (Bethesda). 2013 Oct 11;3(12):2233–8. doi: 10.1534/g3.113.008847 (PMC3852385; doi:10.1534/g3.113.008847)
Supplement: Supporting Information [file supp_g3.113.008847_FigureS1.pdf]

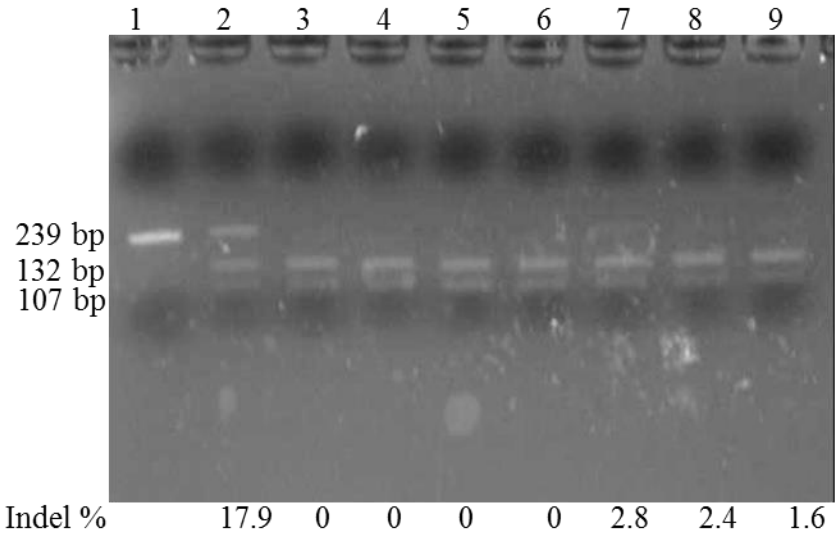

Figure S1 Sequence specificity analysis of CRISPR-Cas system by using different mutated cgRNAs. List of mutant cgRNA is given in Supplementary table 2. Normal and mutant cgRNAs (targeting protospacer 1 of *inox* gene) were co-expressed with Cas9 in suspension cells of *T. aestivum*. Targeted region was amplified, and digested with BsgI restriction enzyme (BsgI restriction site is present at cleavage position in *inox* protospacer1). BsgI could not digest the mutated DNA amplicon. Lane 1 undigested amplicon and lane 2-9 digested with BsgI. Lane 2, mutated with normal cgRNA; 3, wild type amplicon; 4-9, mutated with mutant cgRNA 1, 6, 9, 11, 12 and 13, respectively. Figure shows that mutations at 3' end of target binding region of cgRNA upto 12th bases completely abolished the cleavage activity. However mutations were detected in case of 5' end mutant cgRNA.
