# Supplementary material for: RNA-Guided Genome Editing for Target Gene Mutations in Wheat
Source: G3 (Bethesda). 2013 Oct 11;3(12):2233–8. doi: 10.1534/g3.113.008847 (PMC3852385; doi:10.1534/g3.113.008847)
Supplement: Supporting Information [file supp_g3.113.008847_FileS1.pdf]

File S1

Partial sequence of (A) *inositol oxygenase* (*inox*) and (B) *phytoene desaturase (pds)* genes of wheat, and (C) *pds* gene of *Nicotiana benthamiana* used to decide the target for modification by CRISPR-Cas System. Targeted regions are shown in blue font and PAM in red. Primer used for amplification of gene after editing is shown in green.

(A)

```
                                               Forward primer
                                                ------------->
GCTCGACGGCGGCTTCACCGTGCCGGACTCCAACGCCTTCGGCCACACCTTCAGGGACTACGACGCGGAGTCGGA BASE PAIRS
CGAGCTGCCGCCGAAGTGGCACGGCCTGAGGTTGCGGAAGCCGGTGTGGAAGTCCCTGATGCTGCGCCTCAGCCT 1 TO 75
                                               Protospacer 1
GCGGAAGAAGACGGTGGAGGAGTTCTACAGCGTGAACCACATCAACCAGACGTACGAGTTTGTGCAGCGGATGCG BASE PAIRS
CGCCTTCTTCTGCCACCTCCTCAAGATGTCGCACTTGGTGTAGTTGGTCTGCATGCTCAAACACGTCGCCTACGC 76 TO 150
                    Protospacer 2
GGACGCATACGGGCGGCTGGACAAGACGGAGATGAGCATCTGGGAGTGCATCGAGCTGCTCAACGAGTTCATCGA BASE PAIRS
CCTGCGTATGCCCGCCGACCTGTTCTGCCTCTACTCGTAGACCCTCACGTAGCTCGACGAGTTGCTCAAGTAGCT 151 TO 225

CGACAGTGACCCCGACCTGGACATGCCGCAGATCGAGCACCTCCTCCAAACCGCCGAGGCCATCCGCAAGGACTA BASE PAIRS
GCTGTCACTGGGGCTGGACCTGTACGGCGTCTAGCTCGTGGAGGAGGTTTGGCGGCTCCGGTAGGCGTTCCTGAT 226 TO 300
                                            <-------------
                                              Reverse primer
```

Yellow highlighted region is the site for BsgI used for digestion of amplified DNA.

(B)

```
    Forward primer                                      Protospacer 1
GCTGAGCTTGGTATTAGTGATCGCTTGCAATGGAAGGAACACTCCATGATATTTGCCATGCCAAACAAACCAGGA BASE PAIRS
CGACTCGAACCATAATCACTAGCGAACGTTACCTTCCTTGTGAGGTACTATAAACGGTACGGTTTGTTTGGTCCT 1 TO 75
                                        Protospacer 2
GAATACAGCCGTTTTGATTTTCCAGAGACTTTGCCGGCGCCCTTAAATGGAGTGTGGGCCATACTGAAAAACAAT BASE PAIRS
CTTATGTCGGCAAAACTAAAAGGTCTCTGAAACGGCCGCGGGAATTTACCTCACACCCGGTATGACTTTTTGTTA 76 TO 150

GAAATGCTTACTTGGCCGGAGAAGGTGAAG  BASE PAIRS
CTTTACGAATGAACCGGCCTCTTCCACTTC  151 TO 180

       Reverse primer
```

(C)

```
        Forward primer
GAATTGGTTTTTGCACCTGCAGAAGAGTGGATAAATCGCAGTGACTCTGAAATTATTGATGCTACAATGAAGGAA BASE PAIRS
CTTAACCAAAAACGTGGACGTCTTCTCACCTATTTAGCGTCACTGAGACTTTAATAACTACGATGTTACTTCCTT 1 TO 75
         Protospacer 1                                        Protospacer 2
CTAGCAAAGCTTTTCCCTGATGAAATTTCGGCAGATCAGAGCAAAGCAAAAATATTGAAGTATCATGTTGTCAAA BASE PAIRS
GATCGTTTCGAAAAGGGACTACTTTAAAGCCGTCTAGTCTCGTTTCGTTTTTATAACTTCATAGTACAACAGTTT 76 TO 150

ACTCCAAGGTCTGTTTATAAAACTGTGCCAGGTTGTGAACCCTGCCGGCCCTTGCAAAGATCCCCTATAGAGGGG BASE PAIRS
TGAGGTTCCAGACAAATATTTTGACACGGTCCAACACTTGGGACGGCCGGGAACGTTTCTAGGGGATATCTCCCC 151 TO 225
                                              Reverse primer
```
