# Supplementary material for: RNA-Guided Genome Editing for Target Gene Mutations in Wheat
Source: G3 (Bethesda). 2013 Oct 11;3(12):2233–8. doi: 10.1534/g3.113.008847 (PMC3852385; doi:10.1534/g3.113.008847)
Supplement: Supporting Information [file supp_g3.113.008847_FileS2.pdf]

File S2

Chimeric guide RNA (cgRNA) for targeting (A) *inositol oxygenase* (*inox*) and (B) *phytoene desaturase (pds)* genes of wheat, and (C) *pds* gene of *Nicotiana benthamiana*. (i) cgRNA for protospacer 1, (ii) cgRNA for protospacer 2 and (iii) cgRNA for targeting both protospacer 1 and 2. Target region is given in blue font and guide RNA scaffold in red font. Restriction site SpeI (highlighted in yellow) was used for ligation of the two cgRNAs.

(A)

(i)

NNNNNAGACGTACGAGTTTGTGCAGGTTTTAGAGCTAGAAATAGCAAGTTAAAATAAGGCTA
GTCCGTTATCAACTTGAAAAAGTGGCACCGAGTCGGTGCTTTTNNNNNNNN

(ii)

NNNNNCAAGACGGAGATGAGCATCTGTTTTAGAGCTAGAAATAGCAAGTTAAAATAAGGCTA
GTCCGTTATCAACTTGAAAAAGTGGCACCGAGTCGGTGCTTTTNNNNNN

(iii)

NNNNNAGACGTACGAGTTTGTGCAGGTTTTAGAGCTAGAAATAGCAAGTTAAAATAAGGCTA
GTCCGTTATCAACTTGAAAAAGTGGCACCGAGTCGGTGCTTTACTAGTNNNNCAAGACGGAG
ATGAGCATCTGTTTTAGAGCTAGAAATAGCAAGTTAAAATAAGGCTAGTCCGTTATCAACTT
GAAAAAGTGGCACCGAGTCGGTGCTTTTNNNNNN

(B)

(i)

NNNNNTTTGCCATGCCAAACAAACCGTTTTAGAGCTAGAAATAGCAAGTTAAAATAAGGCTA
GTCCGTTATCAACTTGAAAAAGTGGCACCGAGTCGGTGCTTTTNNNNNNNN

(ii)

NNNNNGGCGCCCTTAAATGGAGTGTGTTTTAGAGCTAGAAATAGCAAGTTAAAATAAGGCTA
GTCCGTTATCAACTTGAAAAAGTGGCACCGAGTCGGTGCTTTTNNNNNN

(C)

(i)

NNNNNGCTTTTCCCTGATGAAATTTGTTTTAGAGCTAGAAATAGCAAGTTAAAATAAGGCTA
GTCCGTTATCAACTTGAAAAAGTGGCACCGAGTCGGTGCTTTTNNNNNNNN

(ii)

NNNNNATCATGTTGTCAAAACTCCAGTTTTAGAGCTAGAAATAGCAAGTTAAAATAAGGCTA
GTCCGTTATCAACTTGAAAAAGTGGCACCGAGTCGGTGCTTTTNNNNNN
