# Supplementary material for: RNA-Guided Genome Editing for Target Gene Mutations in Wheat
Source: G3 (Bethesda). 2013 Oct 11;3(12):2233–8. doi: 10.1534/g3.113.008847 (PMC3852385; doi:10.1534/g3.113.008847)
Supplement: Supporting Information [file supp_g3.113.008847_TableS1.pdf]

Table S1 Plant expression vectors used in the study.

Vector	Detail	Target gene
pCas9	Expression of FLAG tag and NLS containing Cas9 protein.	NA
pTpds1	Expression of cgRNA targeting protospacer 1 of *Nicotiana benthamiana phytoene desaturase (pds)* gene	*N. benthamiana pds* gene
pTpds2	Expression of cgRNA targeting protospacer 2 of *N. benthamiana pds* gene	*N. benthamiana pds* gene
pCtpds1	Co-expression of Cas9 and cgRNA targeting protospacer 1 of *N. benthamiana pds* gene	*N. benthamiana pds* gene
pCtpds2	Co-expression of Cas9 and cgRNA targeting protospacer 2 of *N. benthamiana pds* gene	*N. benthamiana pds* gene
pCinox1	Co-expression of Cas9 and cgRNA targeting protospacer 1 of *T. aestivum inositol oxygenase* (*inox*) gene	*T. aestivum inox* gene
pCinox2	Co-expression of Cas9 and cgRNA targeting protospacer 2 of *T. aestivum inox* gene	*T. aestivum inox* gene
pCwpds1	Co-expression of Cas9 and cgRNA targeting protospacer 1 of *T. aestivum pds* gene	*T. aestivum pds* gene
pCwpds2	Co-expression of Cas9 and cgRNA targeting protospacer 2 of *T. aestivum pds* gene	*T. aestivum pds* gene
pCinox12	Co-expression of Cas9 and duplex cgRNAs targeting protospacer 1 and 2 of *T. aestivum pds* gene	*T. aestivum pds* gene
pCpin1	Co-expression of Cas9 and duplex cgRNAs targeting protospacer 1 of both *inox* and *pds* gene of *T. aestivum.*	*T. aestivum inox* and *pds* gene
