# Supplementary material for: RNA-Guided Genome Editing for Target Gene Mutations in Wheat
Source: G3 (Bethesda). 2013 Oct 11;3(12):2233–8. doi: 10.1534/g3.113.008847 (PMC3852385; doi:10.1534/g3.113.008847)
Supplement: Supporting Information [file supp_g3.113.008847_TableS2.pdf]

Table S2 Specificity analysis of cgRNA. Table shows average mutation percentage at targeted locus with different mutant cgRNA.

		Sequence	Average mutation (%)
Normal cgRNA		AGACGTACGAGTTTGTGCAG	17.9
Mutant cgRNA	1	AGACGTACGAGTTTGTGCAA	0
	2	AGACGTACGAGTTTGTGCGA	0
	3	AGACGTACGAGTTTGTGGCA	0
	4	AGACGTACGAGTTTGGTGCA	0
	5	AGACGTACGGAGTGCAGTTT	0
	6	AGACGTACTAGTTTGTGCAG	0
	7	AGACGTACGAGTTTGTGAAG	0
	8	AGACGTACGAGTTTGTCTAG	0
	9	AGACGTACAAGTTTATGCAG	0
	10	AGACGTACGAGTGGGTGCAG	0
	11	TGACGTACGAGTTTGTGCAG	2.8
	12	CAACGTACGAGTTTGTGCAG	2.4
	13	CAGCGTACGAGTTTGTGCAG	1.6
	14	AGACAGTCGAGTTTGTGCAG	0
	15	AGACTGCAGAGTTTGTGCAG	0
